# The pharmacological and hormonal therapy of hot flushes in breast cancer survivors

**DOI:** 10.1007/s12282-015-0655-2

**Published:** 2015-10-24

**Authors:** Iwona Wiśniewska, Bożena Jochymek, Monika Lenart-Lipińska, Mariusz Chabowski

**Affiliations:** Department of Radiotherapy, Lower Silesian Oncology Center, Wroclaw, Poland; Department of Radiotherapy, Center of Oncology - Maria Skłodowska Curie Memorial Institute, Branch in Gliwice, Gliwice, Poland; Department of Endocrinology, Department of Laboratory Diagnostics, Medical University of Lublin, Lublin, Poland; Department of Clinical Proceedings, Faculty of Health Science, Wroclaw Medical University, 5 Bartla Street, 51-618 Wroclaw, Poland

**Keywords:** Hot flushes, Breast cancer, Menopause, Tamoxifen, Chemotherapy

## Abstract

The side effects of oncological treatment, which appear during or after therapy, are sometimes very annoying for patients and are not adequately treated by physicians. Among the symptoms experienced by breast cancer patients are hot flushes, which result from a natural or cancer therapy-induced menopause. The intensity of hot flushes in breast cancer patients may be more severe than those experienced by women undergoing a natural menopause. Taking into account the incidence of breast cancer and long-lasting hormone-suppression therapies, the problem of hot flushes will affect many women. Hormonal replacement therapy, the most effective therapeutic means for alleviating hot flushes, is usually contraindicated for breast cancer patients. For intense and severe hot flushes, pharmacological treatment using agents from a group of selective serotonin reuptake inhibitors and serotonin and norepinephrine reuptake inhibitors such as venlafaxine or citalopram may be introduced. Other agents from different pharmacological groups, such as clonidine, gabapentin, or pregabalin, have also proved to be effective in treating hot flushes. The efficacy of phytoestrogens has not been proven in randomized clinical trials. The importance of the placebo effect in decreasing vasomotor symptoms has also been reported in many research papers. Educating breast cancer patients in lifestyle changes which decrease the frequency and intensity of vasomotor symptoms can offer significant help too. This paper reviews the current state of research in order to assess the options for the treatment of hot flushes in breast cancer survivors.

## Introduction

In recent years, it has been observed that oncological treatment has become increasingly successful, which is reflected in the prolonged life expectancy of patients. The side effects of oncological therapies which appear during or after treatment seem not to be a problem that doctors are fully aware of. One of the major oncological problems in our society is breast cancer, with an incidence of 16,000 new cases per year in Poland (according to data from 2010). Nowadays, the peak incidence is between 50 and 69 years of age [1]. In this period, most women also undergo the menopausal period, which is difficult for them, as a consequence of the gradual decrease in gonadal function with all its inherent complications. In females treated for breast cancer who had regular menstrual cycles before the oncological therapy, the premature menopause is induced by chemotherapy and hormonotherapy.

Due to the increase in the incidence of breast cancer, the aging of the Polish population, and the tendency of prolonging the time of hormonotherapy even up to 10 years, it can also be assumed that an increasing number of patients are suffering from menopausal symptoms. One of the characteristic menopausal symptoms (apart from the cessation of menstrual cycles) is vasomotor symptom. The term vasomotor symptoms or hot flushes defines the subjective sensation of sudden heat, which is usually most intense over the face, neck, and chest. Hot flushes are often accompanied by a reddening of the face, followed by intensive sweating and even chills. Palpitations, anxiety, and night sweats are also very frequent symptoms. The duration of the symptoms, their frequency, and intensity depend on the individual predispositions of females. In a general population, hot flushes occur in even 75 % of menopausal women [2, 3]. These symptoms interfere with daily life and have a detrimental effect on its quality.

## The pathogenesis of hot flushes

Despite intensive research conducted for many years, the pathophysiology of hot flushes still remains unclear. It seems that the dysfunction of the thermoregulatory center, which is located in the preoptic area of the hypothalamus, plays a key role in this mechanism. The thermoregulatory center maintains the core body temperature within certain homeostasis defined by thermoregulatory thresholds. Sweating occurs when the core temperature of the body increases above the upper threshold of the thermoregulatory zone, whereas chills occur when the core temperature falls below the lower threshold of the thermoregulatory zone [4]. Freedman’s research revealed that women who experience hot flushes had a narrower thermoregulatory zone, which led to a greater likelihood of crossing these thresholds and developing vasomotor symptoms. Increasing estrogen deficiency in the menopausal period, which interferes with the hypothalamic regulatory systems functioning with the use of norepinephrine, serotonin, testosterone, and endorphins, may also contribute to disturbances in the homeostasis of the thermoregulatory center. The result of these interactions triggers the process of hot flushes with the response of the autonomic nervous system and the releasing of hormones [4–6].

## Vasomotor symptoms in women treated due to breast cancer

The oncologic treatment of patients with breast cancer is multidisciplinary and consists of surgical procedure (breast conserving therapy or a radical mastectomy) and adjuvant treatment which includes radiotherapy and systemic therapy. The systemic therapy is individually designed for the patient depending on the subtype of the breast cancer and the clinical and pathologic features of the disease [7]. Chemo- and hormonal therapies induce side effects which include, among others, a premature menopause with its consequences. The incidence of cancer therapy-induced menopause depends on the patients’ age, the model of multi-drug chemotherapy, and its cumulative dose [4, 8]. In 2010, Mar Fan published the results of a prospective study which compared the menopausal symptoms in two groups of patients. One group consisted of patients with a chemotherapy-induced menopause and the second group included women who had gone through a natural menopause. The study confirmed that breast cancer patients experienced significantly more moderate/severe hot flushes than control women undergoing a natural menopause (51 vs 19 %) [9]. In the therapy of breast cancer, which is a hormone dependent cancer, tamoxifen and a group of drugs called aromatase inhibitors (AI) hold a very important position. The indication for the use of these drugs is the confirmed expression of steroid receptors in the cancer cells. Tamoxifen can be applied in all breast cancer patients at every stage of cancer therapy and also as a prophylaxis. Tamoxifen belongs to the group of agents which are called selective estrogen receptor modulators (SERM) and exerts its antineoplastic action by blocking estrogen receptors (ER) in cancer cells. In this way, tamoxifen prevents the interaction of endogenous estrogens with ER. In postmenopausal females, estradiol is synthesized almost exclusively in peripheral tissues from the conversion of androstenedione to estrone with the use of aromatase. The mechanism of the action of AI in the treatment of postmenopausal patients is to inhibit peripheral estrogen synthesis [10].

The clinical trials evaluating tamoxifen revealed that even up to 80 % of females experienced hot flushes during therapy with this agent, and 30 % of patients defined these symptoms as severe [3]. Despite the high efficacy of tamoxifen, the harmful side effects have been identified in previous studies as a significant reason for not persisting with treatment for 16–30 % of breast cancer patients [11]. AI used in postmenopausal patients also induce vasomotor symptoms. In randomized trials comparing the efficacy of AI with tamoxifen, it has been revealed that hot flushes induced by AI were less severe. In an ATAC study, 35.7 % of patients treated with anastrozole experienced vasomotor symptoms in comparison to 40.9 % of patients treated with tamoxifen. Similarly, in BIG 1–98 study, 33.5 % of females taking letrozole suffered hot flushes compared to 38 % of patients treated with tamoxifen [12].

## Hormonal replacement therapy and breast cancer

Hormonal replacement therapy (HRT) is the most efficient means for alleviating menopausal symptoms, not only hot flushes [10]. Many trials have been carried out evaluating HRT in healthy postmenopausal women. One of them was the big American randomized trial, the Women’s Health Initiative (WHI), which assessed the benefits of estrogen plus progestin therapy among healthy postmenopausal women in comparison to the placebo group. The results of the WHI demonstrated a 26 % increase in the risk of breast cancer in the group treated with HRT compared to those diagnosed in the placebo group [13]. The increased risk of breast cancer in healthy postmenopausal women treated with HRT was also revealed in the Million Women Study. It was confirmed that the risk of breast cancer was dependent on the duration of the treatment and the type of HRT used [14].

Trials have been also conducted addressing the issue whether HRT is safe for women who have previously had breast cancer. One of them is the Swedish randomized trial, the hormone replacement therapy after breast cancer (HABITS), which was stopped when the initial analysis revealed a doubly increased risk of recurrence after hormone replacement therapy in breast cancer survivors (locoregional recurrence, distant metastases, or cancer of the opposite breast). After extended follow-up, there was significantly increased risk of new breast cancer occurring in women who took HRT in comparison to the placebo group (22.2 vs 8 %, respectively) [15]. After the results of the HABIT trial, the second Swedish randomized trial—Stockholm trial was also stopped in 2003, even though the initial analyses showed no increased risk of breast cancer recurrence among women taking HRT [16]. Data from the 10-year follow-up to the Stockholm randomized trial are currently available. The authors of the paper demonstrated that in both studies among women on HRT, no increased mortality due to breast cancer or other malignancies was found. The increased recurrence in HABITS has been attributed to higher progestin exposure [17]. However, the negative relationship between HRT and breast cancer has been confirmed in many trials. These studies concerned both combined HRT and only estrogen or progestational agents. In conjunction with these results, HRT is contraindicated in females after breast cancer therapy [4, 18].

## Pharmacological treatment

### Antidepressants agents: SSRIs and SNRIs

Selective serotonin reuptake inhibitors (SSRIs) and serotonin and norepinephrine reuptake inhibitors (SNRIs) are the classes of drugs which are used for hot flushes therapy in breast cancer patients and their efficacy has been proven in randomized clinical trials [3, 18].

A daily dose of 10 mg of paroxetine, 20 mg of fluoxetine, and 10–20 mg of citalopram all belong to SSRIs, which have a great body of evidence for their beneficial effect in the treatment of hot flushes. Venlafaxine, recommended with a daily dose from 37.5 to 75 mg, is believed to be the most important agent in the group of SSRIs. The use of venlafaxine has been evaluated in five randomized clinical trials: in two placebo-controlled trials and in three trials with clonidine.

Studies show that the use of SSRIs and SNRIs significantly reduced the frequency and severity of hot flushes in breast cancer patients by 14–58 % in comparison to the placebo. However, the most common side effects caused by these drugs include dry mouth, headaches, constipation, nausea, and loss of appetite [18].

What is more, SSRIs and SNRIs are inhibitors of the cytochrome P450 2D (CYP2D6) pathway, an important enzyme which metabolizes tamoxifen to its active form—endoxifen. Fluoxetine and paroxetine are strong P450(CYP)2D6 inhibitors and these drugs significantly decrease plasma endoxifen concentration and should not be administered along with tamoxifen [18–20]. Citalopram is considered to be a much weaker P450(CYP)2D6 inhibitor, which gives only a slight decrease in plasma endoxifen concentration, which is probably of no clinical relevance [3]. Venlafaxine also turned out to be a weak P450(CYP)2D6 inhibitor. The data show no influence on endoxifen concentration in patients taking tamoxifen. In conclusion, venlafaxine may be used in the treatment of hot flushes in breast cancer patients taking tamoxifen [3, 18].

### Other drugs

Clonidine, an antihypertensive agent, has been reported as effective in the treatment of hot flashes in postmenopausal women based on a daily dose 0.1 mg [21].

In randomized clinical trials, the efficacy of antiepileptic agents such as gabapentin and pregabalin has also been proven. The recommended initial daily dose of gabapentin of 300 mg, gradually increased to 900 mg, and pregabalin with a dose of 50–150 mg, resulted in a significant reduction in the frequency of hot flushes [3, 18].

It has been reported that a dose of 400 IU of vitamin E twice daily may have a marginally beneficial influence on treating hot flushes [3, 22].

### Phytoestrogens

Phytoestrogens are plant-derived compounds, closely related to steroids—phytosterols and isoflavones. In their structure, phytoestrogens are similar to the female steroid hormones produced by the ovaries and adrenal glands, and they cause both estrogenic and antiestrogenic activity in the human body. The main source of phytoestrogens and isoflavones in a diet is soy, chickpeas, mung beans, and alfalfa sprouts. These compounds can also be found in small amounts in cereals, rice, onions, and garlic. Lignans, which are present in flax seeds, sunflower seeds, olives, cherries, sesame seed oil, garlic, and tea, also show weak estrogen properties [23]. The efficacy of phytoestrogens in hot flushes treatment in breast cancer patients has been studied in many randomized clinical trials. These studies compared the efficacy of different formulas containing various phytoestrogens and isoflavons, including soya and flaxseed products with the placebo. None of these trials demonstrated the superiority of phytoestrogens in reducing hot flushes in comparison to the placebo [24–28]. Tempfer’s metaanalysis assessed the side effects caused by phytoestrogens used in the menopausal period for the treatment of climacteric syndrome. The authors revealed, comparing the treatment with the placebo, that phytoestrogens are safe but may moderately elevate rates of gastrointestinal side effects [29].

In alternative medicine, black cohosh is recommended as a natural treatment for menopausal symptoms. Current studies conducted in breast cancer patients treated with black cohosh reveal that black cohosh were not effective in reducing hot flushes in this group of females. The available data also do not support an association between black cohosh and an increased risk of breast cancer, as was suggested in previous observations [30].

What is interesting is that in many clinical trials controlled with a placebo, a substantial placebo effect has been shown in the treatment of hot flushes. It has been demonstrated that the placebo effect can reduce hot flushes by even 50 %. Therefore, the placebo effect should be always seriously taken into account [12].

## Patient education

Informing the cancer patients of both the side effects of the therapy as well as the methods of their prevention plays a crucial role in the work of healthcare professionals. In the literature, the emphasis has been on the importance of patient education in life style changes, which decrease the frequency and feeling of vasomotor symptoms. Avoiding all situations which can trigger hot flushes has been suggested, including such triggers as stress, caffeine, hot spices, hot rooms, and hot baths. It has been also advised that patients should maintain a low core body temperature through dressing in layers, consuming cool drinks and food, and using a fan as needed. Stopping smoking, maintaining a healthy weight (BMI up to 27 kg/m^2^), regular physical activity, and relaxation techniques are also very important [3, 4, 31, 32].

## Conclusions

In recent years, numerous studies have been carried out on the efficacy of pharmacological and nonpharmacological methods of treatment of vasomotor symptoms in the course of a natural or breast cancer chemo- or hormonotherapy-induced menopause. Menopausal symptoms, including hot flushes, are reported by the majority of breast cancer patients and they contribute to a poor quality of life. Taking into account the increasing incidence of breast cancer and existing indications for extended treatment with tamoxifen, this problem will affect more and more breast cancer survivors. For severe hot flushes, the above mentioned pharmacological treatment may be introduced. What is important is that the approach to the patients should always be individually designed, after a careful assessment of the benefits and side effects of the therapy being suggested. All the patients should be educated on the simple but effective methods of minimizing the intensity and frequency of hot flushes symptoms in everyday life.
